# Orthodontic incisor retraction caused changes in the soft tissue chin area: a retrospective study

**DOI:** 10.1186/s12903-020-01099-2

**Published:** 2020-04-15

**Authors:** Wenxin Lu, Xu Zhang, Li Mei, Pengfei Wang, Jiarong He, Yu Li, Zhihe Zhao

**Affiliations:** 1 State Key Laboratory of Oral Diseases, National Clinical Research Center for Oral Diseases, Department of Orthodontics, West China Hospital of Stomatology, Sichuan University, 14 Renmin South Road Third Section, Chengdu, 610041 China; 2 Discipline of Orthodontics, Department of Oral Sciences, Faculty of Dentistry, Sir John Walsh Research Institute, University of Otago, Dunedin, New Zealand

**Keywords:** Bimaxillary protrusion, Soft tissue change, Tooth extraction, VTO

## Abstract

**Background:**

To investigate the area and morphological changes around the soft tissue chin after orthodontic incisor retraction.

**Methods:**

Fifty-nine female adults with bimaxillary protrusion requiring extraction of four premolars were included in the study. Cephalograms were taken before (T0) and after (T1) orthodontic treatment. The soft tissue changes, including the area, thickness and morphology were measured. Paired-t tests were performed for statistical comparisons. Pearson correlation analyses and backward multivariate regression analyses were used to identify the relationship between the soft tissue changes and incisor retraction.

**Results:**

Following the incisor retractions (5.35 ± 1.79 mm and 4.42 ± 1.62 mm for the upper and lower, respectively), there was a significant increase in the soft tissue thickness of L1c-LL (0.64 ± 1.67 mm, *P* = 0.025) and Pog-Pog’ (0.44 ± 1.10 mm, *P* = 0.022), and a significant decrease in the soft tissue thickness of B-B′ (1.21 ± 1.34 mm, *P* <  0.01). Changes in the area of soft tissue chin and lower lip were not statistically significant (*P* > 0.05). Pearson coefficient between the thickness changes of B-B′ and the retraction of lower incisors was − 0.376. The multiple correlations between the soft tissue thickness changes and incisor retractions were *Y = 1.02–0.42a + 0.42b* for L1c-LL, and *Y = 0.17–0.31b* for B-B′.

**Conclusions:**

The orthodontic incisor retraction could cause soft tissue thickness changes (i.e. an increase in L1c-LL and Pog-Pog’ and a decrease in B-B′) without area changes.

## Background

Facial appearance plays an important role in an individual’s daily life, social interactions, self-esteem and psychological well-being [1]. It has been found that the public increasingly pays attention to the lips and soft tissue chin rather than to other orofacial structures when assessing facial esthetics [2]. The soft tissue chin in patients with dental protrusion, however, is strained resulting in undermined chin prominence [3]. The orthodontic treatment of bimaxillary protrusive patients usually involves the extraction of premolars and retraction of anterior teeth, which results in increased chin prominence and improved facial profiles [4]. Several studies and clinical trials have shown that these changes of soft tissue chin were mainly due to the redistribution or reshaping of the soft tissues around the chin area following the retraction of incisors [5].

Most of the previous studies used cephalometry and investigated the ratio between the amount of incisor retractions and soft tissue changes. For example, some researchers have reported the predictive ratios for lower lip change along with the mandibular incisor advancement ranging from 0.26 to 0.85:1, and 1:1 for the change of soft tissue pogonion to the advancement of hard tissue pogonion [6]. Others have suggested that there were significant individual variations for the changes in the soft tissue following the extraction treatment because of many influencing factors, such as soft tissue thickness, soft tissue areas, the underlying skeletal patterns [7], the soft tissue remodeling during orthodontic treatment and the strain of soft tissue upon the anterior teeth. Though it is now possible to simulate soft tissue changes for patients with dental protrusion and extraction treatment by using some visual treatment objective (VTO) software, it is still difficult to accurately predict the soft tissue changes in the chin area following the orthodontic incisor retraction [8–10].

Although the 3-dimensional cone-beam computed tomography (CBCT) has advantages for researching the hard and soft tissues changes following orthodontic treatment, the conventional 2-dimensional cephalogram is still of great clinical importance and commonly used in orthodontic diagnosis and treatment planning, due to the limitations of CBCT, such as high cost and radiation exposure [11]. An accurate prediction of soft tissue changes following incisor retraction using cephalograms has been considered to be clinically convenient and relevant for orthodontic treatment planning and doctor-patient communication [12].

The study aimed to measure the area and morphological changes in soft tissues around the chin following orthodontic incisor retraction in patients with bimaxillary dental protrusion, and to investigate the relationship between these soft tissue changes and the incisor retraction. The hypothesis was that the area around the soft tissue chin would enlarge and reshape following the incisor retraction.

## Methods

### Subjects

The study was designed as a retrospective observational study. Ethical approval for the study was obtained from the Ethics Committee of the West China Hospital of Stomatology, Sichuan University (WCHSIRB-ST-2017-131). Written informed consent was obtained from each participant.

A total of 59 female adult patients (mean age 23.50 ± 2.15 years, range 18–39 years) were recruited in the study (Table 1). A sample size calculation was undertaken using the nQuery Adviser software package (Version 7.0; Statistical Solutions, Cork, Ireland). The pilot study [13] estimated that the effect size was 0.40. Based on a significance level of alpha 0.05, the sample size was calculated to achieve an 80% power. The sample size calculation showed that 39 subjects were necessary.

**Table 1:** Patients’ age and weight at pre-treatment (T0) and post-treatment (T1)

Measurement	T0		T1		Difference (T1-T0)	
	Mean	SD	Mean	SD	Mean	SD
Age (year)	23.5 (18–39)	2.15	25.7 (20–42)	2.58	2.27	0.57
Weight (kg)	55.68	2.81	55.08	2.60	−0.60	1.38

Inclusion criteria were: (1) young female adults (18–40 years old); (2) Skeletal Class I, Angle Class I bimaxillary dental protrusive malocclusion and crowding less than 4 mm in both arches with normal overjet and overbite; (3) wore stainless steel brackets (Victory Series, 3 M Unitek, Monrovia, California, USA) with extraction of four premolars and anchorage reinforcements, including transpalatal arch, Nance button, headgear, and mini-screw. Exclusion criteria were: (1) previous surgery on the maxilla, mandible or chin; (2) history of craniofacial defects or syndromes, e.g. cleft lip and palates; (3) body weight change more than 5% during the orthodontic treatment.

### Cephalometric analysis

The lateral cephalograms were taken before (T0) and after (T1) orthodontic treatment using a Cephalometer (Veraviewepocs, Morita, Kyoto, Japan). Each subject was positioned with the sagittal plane at a right angle to the path of the X-rays, the Frankfort plane paralleled to the horizontal, the teeth in centric occlusion, and the lips lightly closed. Dolphin Imaging software version 11.0 (Patterson Dental Supply, St. Paul, MN) was used for the cephalometric tracing and analysis.

**Fig. 1:**
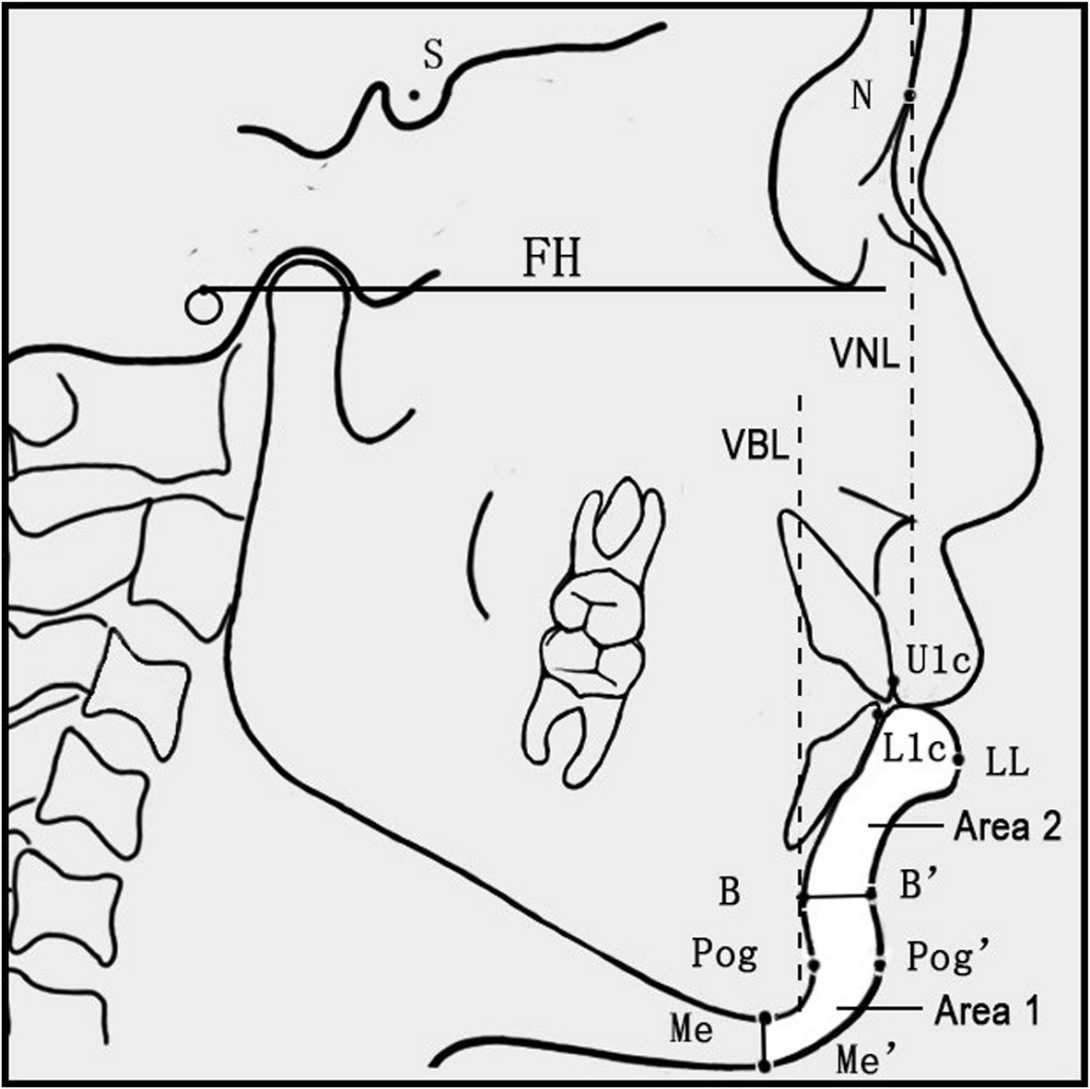
Landmarks, reference planes and cephalometric measurements used in the study

The Frankfort Horizontal (FH) plane was used as the horizontal reference plane (Fig. 1). Two vertical lines perpendicular to the FH plane, one passing the N point (VNL) and the other passing the B point (VBL), served as the vertical reference lines for the maxillary and mandibular evaluations, respectively. Based on the literature [14, 15], the variables, including related cephalometric measurements, the amount of incisor retractions, two soft tissue areas (soft tissue chin and lower lip) and three soft tissue thicknesses (L1c-LL, B-B′ and Pog-Pog’) were illustrated in Fig. 1 and Table 2. The primary outcome variables were the changes of soft tissue area and thickness in response to the incisor retraction between pre-treatment and post-treatment. The secondary outcome variables included the changes of related cephalometric measurements between pre-treatment and post-treatment. The cephalometric radiographs before and after the treatment were superimposed on the cranial base to ensure consistency.

**Table 2:** Landmarks, reference planes and measurements used in the study

	Definition
***Landmarks***	
N	Nasion: most anterior point of the frontonasal suture where the lines of the glabella profile meet those of the nasal bones
S	Sella: Center of bony contour of sella turcica
P	Porion: the midpoint of the upper contour of the metal ear rod of the cephalometer (machine porion)
Or	Orbitale: the lowest point on the inferior margin of the orbit
A	Most concave point between anterior nasalspine and superior prosthion
U1	The tip of the maxillary central incisors
U1c	The most anterior point of clinical crown of maxillary central incisors
L1	The tip of the mandibular central incisors
L1c	The most anterior point of clinical crown of mandibular central incisors
B	Most concave point on mandibular symphysis
Pog	Pogonion: the most anterior point on the osseous contour of the chin
Me	Menton: the most inferior midline point on the mandibular symphesis
LL	The most anterior point of the lower lip based on the reference plane
Stmi	Uppermost point on vermilion border of lower lip
B′	The innermost point on the contour of the soft-tissue between the lower lip and the soft tissue chin
Pog’	Soft tissue pogonion: the most prominent point on the chin based on the reference plane
Me’	Soft tissue menton: the lowest point on the contour of the soft tissue chin
***Reference planes***	
FH	Frankfurt Horizontal plane formed by Portion and Orbitale
MP	Mandibular plane through Me and the lower margin of mandibular angle
VBL	A line passing through the B point and perpendicular to the FH plane serving as the vertical reference for the mandibular evaluations
VNL	A line passing through the N point and perpendicular to the FH plane serving as the vertical reference for the maxillary evaluations
***Cephalometrics analysis index***	
ANB (°)	The ANB angle shows the difference between the maxilla and mandible
SNA (°)	The SNA angle is used to establish the relationship of the maxilla to the cranial base
SNB (°)	The SNB angle is used to establish the relationship of the mandible to the cranial base
U1-L1 (°)	Upper and lower central incisors Angle: the intersection Angle of the long axis of the upper and lower central incisors, representing the relative protrusion of the upper and lower central incisors
U1-NA (°)	The intersection Angle between the long axis of the upper central incisor and the NA line, representing the inclination of the upper central incisor
L1-NB (°)	The intersection Angle between the long axis of the lower central incisor and NB line, representing the inclination of the lower central incisor
SN-MP (°)	The Angle between the mandibular plane and the SN plane
FMA (°)	The Angle between the Frankfurt Horizontal plane and the Mandibular plane
Stmi-Me’ (mm)	Vertical distance between the landmarks of Stmi and Me’
***Soft tissue area measurements***	
Area 1 (cm^2^)	The area of soft tissue chin from the border of B-B′ till the border of Me-Me’
Area 2 (cm^2^)	The area of lower lip till the border of B-B′
***Soft tissue thickness measurements***	
L1c-LL (mm)	Distance between the landmarks of L1c and LL
B-B′ (mm)	Distance between the landmarks of B and B′
Pog-Pog’ (mm)	Distance between the landmarks of Pog and Pog’

The amount of upper incisor retraction was the change of the horizontal distance between U1c and VNL before and after treatments. The amount of lower incisor retraction was the change of the horizontal distance between L1c and VBL before and after treatments. U1c and L1c were the most anterior points of the clinical crown of the upper and lower incisors, respectively (Fig. 1). Soft tissue thicknesses were the direct distances between the landmarks of L1c to LL, B to B′ and Pog to Pog’. The areas of soft tissue chin and lower lip (i.e. Area 1 and 2 in Fig. 1) were measured in mm^2^ using a digital planimeter on Auto CAD 2016 (Autodesk, Inc. Saint Rafael, CA, USA) [16].

Both intra- and inter-operator reliabilities were evaluated using the Bland-Altman method and intraclass correlation coefficients. Briefly, thirty cephalograms were randomly selected and measured by two independent dental investigators. Each investigator repeated the measurements after 1 week. The inter-rater reliability was excellent (correlation coefficient was 0.90). The intra-rater reliability was excellent (correlation coefficients for the two investigators were 0.95 and 0.91).

### Statistical analysis

SPSS 19.0 (SPSS Inc., Chicago, IL, USA) was used for statistical analysis of the data. The skewness and kurtosis statistics demonstrated normal distributions. Paired-t tests were performed for statistical comparison of the soft tissue changes before and after the incisor retraction. Pearson correlation analysis and backward multivariate regression analysis were used to identify the relationships between the soft tissue changes and the incisor retractions. Multivariate regression models were established and derived as: *Y = Constant + a + b*, where “*Y*” was the soft tissue thickness change, and “*a*” and “*b*” were the coefficient values for the retraction of upper incisors and lower incisors, respectively.

## Results

### Incisor retraction

The amount of upper incisor retraction was 5.35 ± 1.79 mm (95% Confidence Interval (CI): 4.75–5.95 mm). The amount of lower incisor retraction was 4.42 ± 1.62 mm (95% CI: 3.88–4.96 mm). The changes of related cephalometric measurements along with incisor retraction were shown in Table 3.

**Table 3:** Changes of cephalometric measurements between pre-treatment (T0) and post-treatment (T1)

Measurement	T0		T1		T1-T0		*P*-value
	Mean	SD	Mean	SD	Mean	SD	
ANB (°)	4.18	1.40	3.72	1.74	−0.45	1.11	0.02
SNA (°)	83.35	3.37	82.81	3.80	− 0.54	1.48	0.12
SNB (°)	79.17	3.11	79.09	3.74	− 0.08	1.50	0.19
U1-L1 (°)	109.39	7.32	131.77	10.00	22.38	10.87	< 0.01
U1-NA (°)	31.28	5.58	19.68	7.49	−11.60	6.23	< 0.01
L1-NB (°)	35.37	4.15	25.16	5.34	−10.21	7.20	< 0.01
SN-MP (°)	33.20	5.92	34.14	5.33	0.94	2.73	0.05
FMA (°)	27.15	5.41	27.37	5.32	0.22	3.03	0.67
Stmi-Me’ (mm)	44.14	3.03	44.38	2.50	0.24	1.95	0.47

### Soft tissues area change

No statistically significant difference of area change was found in the soft tissue chin (Area 1, T1-T0 = 0.14 ± 0.50 cm^2^, *P* = 0.08) or in the lower lip (Area 2, T1-T0 = − 0.03 ± 0.40 cm^2^, *P* = 0.69) with the incisor retraction (Table 4).

**Table 4:** Changes in the soft tissue area between pre-treatment (T0) and post-treatment (T1)

Measurement	T0		T1		T1-T0		*P*-value
	Mean	SD	Mean	SD	Mean	SD	
Area 1 (cm^2^)	2.86	0.44	3.01	0.55	0.14	0.50	0.08
Area 2 (cm^2^)	2.17	0.57	2.14	0.62	−0.03	0.40	0.69
Area 1 + 2 (cm^2^)	5.03	0.83	5.15	0.99	0.12	0.76	0.35

### Soft tissue thickness change

Following the incisor retraction, there was a significant increase in the soft tissue thickness of L1c-LL (0.64 ± 1.67 mm, *P* <  0.05) and Pog-Pog’ (0. 44 ± 1. 10 mm, *P* <  0.05), and a significant decrease in the soft tissue thickness of B-B′ (1.21 ± 1.34 mm, *P* <  0.01) (Table 5).

**Table 5:** Changes in the soft tissue thickness between pre-treatment (T0) and post-treatment (T1)

Measurement	T0		T1		T1-T0		*P*-value
	Mean	SD	Mean	SD	Mean	SD	
L1c-LL (mm)	10.36	1.70	11.00	1.61	0.64	1.67	0.03
B-B′ (mm)	12.09	1.64	10.89	1.55	−1.21	1.34	< 0.01
Pog-Pog’ (mm)	10.44	1.69	10.89	1.64	0.44	1.10	0.02

### Relationship between the soft tissue thickness change and incisor retraction

Pearson correlation analysis showed a negative correlation between the thickness change of B-B′ and the lower incisor retraction (correlation coefficient = − 0.376, *P* <  0.05). No statistically significant correlation was found between the incisor retraction and the thickness change of L1c-LL and Pog-Pog’ (Table 6).

**Table 6:** Pearson correlation coefficients between the incisor retraction, ANB, SN-MP and soft tissue thickness changes

Soft tissue thickness changes	Upper incisor retraction		Lower incisor retraction		ANB		SN-MP	
	Correlation coefficient	*P*-value	Correlation coefficient	*P*-value	Correlation coefficient	*P*-value	Correlation coefficient	*P*-value
L1-LL	−0.22	*P* = 0.19	0.16	*P* = 0.34	0.230	*P* = 0.17	0.126	*P* = 0.46
B-B′	−0.26	*P* = 0.12	− 0.38	*P* < 0.05	0.080	*P* = 0.64	0.200	*P* = 0.23
Pog-Pog’	−0.05	*P* = 0.79	− 0.04	*P* = 0.83	0.273	*P* = 0.10	0.122	*P* = 0.47

### Multivariate regression analysis

The multivariate regression analysis revealed that the multiple correlations between the soft tissue thickness changes and incisor retractions were *Y = 1.02–0.42a + 0.42b* for L1c-LL, and *Y = 0.17–0.31b* for B-B′ (“*Y*” was the soft tissue thickness change, “*a*” and “*b*” were the retractions of upper incisors and lower incisors, respectively) (Table 7).

**Table 7:** Multivariate regression analysis of the incisor retraction and soft tissue thickness changes

Soft tissue thickness changes	R^2^	*P*-value	Constant	*a*	*b*
L1c-LL	0.164	0.047	1.02	− 0.42	0.42
B-B′	0.142	0.022	0.17	–	−0.31

## Discussion

The accuracy of prediction in the changes of the soft tissue chin area after orthodontic treatment using cephalogram is still poorly understood. The relationship between incisor movement and soft tissue change is still controversial. This may be because the soft tissue changes can be affected by not only incisor movement but also many other factors, such as dentofacial morphology, age, sex, ethnicity, soft tissue thickness and tension, and the technologies used for estimation [17, 18]. To minimize the influence of sex on soft tissue changes following the incisor movement, only female patients were included in this study.

Some studies have reported that the ratio between lip change and incisor retraction ranged from 1:0.45 to 1.25 for the upper lip, and from 1:1.2 to 1:6.2 for the lower lip in nongrowing patients with bimaxillary protrusion [19, 20]. In addition to the changes of lip position, the incisor retraction could also induce soft tissue thickness changes [19, 21]. In the current study, after incisor retraction (5.35 ± 1.79 mm and 4.42 ± 1.62 mm for the upper and lower, respectively), the soft tissue thickness of L1c-LL, B-B′ and Pog-Pog’ increased 0.64 ± 1.67 mm, − 1.21 ± 1.34 mm and 0.44 ± 1.10 mm, respectively. This may be due to the reduced tension and deformation of the muscles around the lower lip, such as orbicularis oris and mentalis [14]. Another reason may be the thickness measured in the study, in fact, included the real thickness of the lower lip as well as the labial vestibule, which may also re-arrange after incisor retraction [17]. The rotation of the mandible could also influence the tension of soft tissues around the chin area. However, no statistically significant change existed in SN-MP and Stimi-Me’ before and after treatments. Moreover, although ANB decreased 0.45 ± 1.11°after treatments, no correlation was found between the change of ANB and the change of soft tissue thicknesses. One possible explanation is that the change of ANB was clinically inconspicuous in our study.

As for the thickness change of the lower lip, Kuhn found that the lower lip thickness decreased about 2.5 mm in patients with extraction treatments [22]. Some studies, on the other hand, found that the thickness of the lower lip increased [23]. They attributed these lip thickness changes to the muscular tension and deformation of lips, as well [17, 24, 25]. Nevertheless, many studies found no significant change in the lower lip thickness after incisor retractions [22, 26]. The multiple regression analysis in our study showed that the thickness of L1c-LL was affected by the retraction of upper and lower incisors at the same time. Many scholars detected that the upper incisors had effects on the shape and position of the lower lip, probably because the lower lip often covers the upper incisor by a third [2].

The majority of studies found the thickness of B-B′ had decreased [17, 24, 27], which are consistent with our study. The Pearson coefficient showed a negative correlation between the soft tissue thickness change of B-B′ and the retraction of the lower incisor. The average change of soft tissue thickness of B-B′ was less than zero. Thus, the more the retraction, the larger the thickness reduction of B-B′ within limits. Unfortunately, the changes of soft tissue thickness around the chin area following incisor retraction are still inconclusive, especially for the change of Pog-Pog’ which was found to increase, decrease or stay the same after tooth extraction [13].

Most of the previous studies on soft tissue changes were focused on linear or/and angular measurements using conventional two-dimensional cephalograms; a few studies investigated the soft tissue area changes [8, 28]. The changes of soft tissue are complicated; therefore, we need various kinds of data to get a more consummate prediction. The thickness and volume changes of the soft tissue could give us a rounded analysis of a certain area that couldn’t be provided by the changes of merely linear or/and angular measurements. For example, Dai detected the buccal facial depth decreased in adult female patients undergoing extraction by using a three-dimensional structured light scanning system [29]. To measure the volume changes, CBCT scanning is preferable. However, this is not quite applicable so far due to ethical and technique reasons. Thus, measurements of the area on 2D cephalogram remain a practical approach. In this study, besides the linear and angular measurements on the cephalography, incisor retractions, and soft-tissue thicknesses, the areas of soft tissue chin and lower lip were also measured using a digital planimeter [16]. It was found that the muscle tension around the chin region decreased with the degree of maxillary incisor retraction, which might increase the area of the soft tissues around the chin [14]. However, no significant change was found in the areas of the soft tissue chin and lower lip in the study. This may because the soft tissues around the chin region are relatively attached to the basal bone with less mobility. According to this finding, the areas of soft tissue chin and lower lip should be set as invariants in software programming. Based on this rule, a more accurate prediction could be made for the morphological changes of the soft tissue chin combined with the changes of other anatomical landmarks after incisor retractions. Also, if the area of soft tissue chin was found increased after treatments, we would highly suspect that filling material existed in the soft tissue chin.

Apart from the fact that the measurements in the study were two-dimensional and performed on lateral cephalometric radiographs, other limitations of the study may also restrict the generalization of the results, for example, samples were all young female adults and of Asian ethnicity. Studies in the future could include both men and women with a wide range of age and ethnicity, and consider using three-dimensional techniques, such as CT and stereophotogrammetry [29], in order to get a more accurate prediction of the soft tissue changes following different types of tooth movements.

## Conclusions

The area of soft tissue chin and lower lip did not change significantly after orthodontic incisor retraction in female young adult patients with bimaxillary protrusion. Following the incisor retraction, the soft tissue thicknesses of L1c-LL and Pog-Pog’ increased, while in the soft tissue thickness of B-B′ decreased. High-quality and well-designed prospective trials are needed in order to make a more accurate conclusion.

## Data Availability

The datasets used and/or analyzed during the current study are available from the corresponding author on reasonable request.

## Abbreviations

VTO: Visual treatment objective
CBCT: Cone-beam computed tomography
FH: Frankfort Horizontal
